# Kawasaki Disease and Systemic Juvenile Idiopathic Arthritis – Two Ends of the Same Spectrum

**DOI:** 10.3389/fped.2021.665815

**Published:** 2021-04-28

**Authors:** Ellen Go, Mira van Veenendaal, Cedric Manlhiot, Rayfel Schneider, Brian W. McCrindle, Rae S. M. Yeung

**Affiliations:** ^1^The Hospital for Sick Children, Division of Rheumatology, University of Toronto, Toronto, ON, Canada; ^2^University Medical Center Utrecht, Division of Rheumatology, Wilhelmina Children's Hospital, Utrecht, Netherlands; ^3^The Hospital for Sick Children, Division of Cardiology, Labatt Family Heart Centre, University of Toronto, Toronto, ON, Canada

**Keywords:** kawasaki disease, juvenile idiopathic arthritis, systemic juvenile idiopathic arthritis, macrophage activation syndrome, fever, interleukin 1

## Abstract

Kawasaki disease (KD) and systemic juvenile idiopathic arthritis (sJIA) are two distinct systemic inflammatory diseases of childhood. Each diagnosis is based on criteria, but numerous clinical features are overlapping. As no specific diagnostic tests are available, differentiation between both disease entities can be challenging. Here, we describe the disease course of patients with co-diagnosis of both KD and sJIA (KD/sJIA). All our KD (*n* = 1765) and sJIA (*n* = 112) cases were critically reviewed for co-diagnosis of KD/sJIA. Eight KD/sJIA cases were identified and their clinical presentation, treatment regimens, coronary artery outcome and complications are herein described. Each KD/sJIA patient fulfilled diagnostic criteria for KD and for sJIA. Ongoing fever, rash and arthritis were present in each patient. The KD/sJIA patients had recalcitrant KD requiring multiple doses of intravenous immunoglobulin and steroids. Five patients had coronary artery dilatation at KD diagnosis, which resolved in all by 6 weeks. Pericardial effusion was present in 5 patients. One KD/sJIA patient developed macrophage activation syndrome. In conclusion, a small proportion (0.5%) of our KD patients evolved into sJIA, and 7% of our sJIA population presented initially as KD. KD/sJIA patients were characterized by a recalcitrant KD course and a high prevalence of coronary artery dilatation. Patients with co-diagnoses may provide a clue to potentially shared immunopathology in KD and sJIA, leading us to posit that both entities may be part of the same clinical spectrum.

## Introduction

Kawasaki disease (KD) and systemic juvenile idiopathic arthritis (sJIA) are two distinct systemic inflammatory diseases of childhood. Each diagnosis is based on criteria, but numerous clinical features are overlapping. As no specific diagnostic tests are available, differentiation between KD and sJIA can be challenging. Recent research suggests underlying biologic similarities between these two childhood inflammatory diseases with involvement of interleukin-1 beta (IL-1β). We critically reviewed patients with combined diagnoses of KD and sJIA (KD/sJIA) in order to characterize discriminating findings at baseline and provide evidence for their potentially shared immunopathology.

## Materials and Methods

Medical records of all patients diagnosed with either KD (*n* = 1765) or sJIA (*n* = 112) at a tertiary referral center between January 1990 and December 2011 were reviewed. KD/sJIA patients (*n* = 8) were included who both fulfilled the American Heart Association guidelines for KD (1) and the International League of Associations for Rheumatology (ILAR) classification criteria for sJIA (2). Data from the records included demographic information, clinical features, laboratory results, echocardiographic findings, medical treatment and outcome.

Data were summarized as frequencies with percentages, means with standard deviation or median with interquartile range. Data were analyzed using *t*-test and a *P*-value of <0.05 was considered significant.

## Results

### Patient Characteristics and Treatment

In Table 1, we compared baseline characteristics, treatment received and disease complication between KD and KD/sJIA patients. In the KD cohort, median age at diagnosis is 3.1 years (IQR 1.7-5.3), majority of whom were between age range of 1-9 years old (81%) and male (62%). The KD/sJIA patients were diagnosed with KD at median age of 4.7 (IQR 2.1-5.6), mostly male (75%) and no one presented with symptoms at <1 year or >9 years of age. Conjunctival injection was less frequent noted among all the KD features. All of them had rash and half had incomplete KD features, although not statistically significant.

**Table 1:** Baseline features at diagnosis, medication and complication^*^.

	**KD**	**KD/sJIA**	***P-*value^†^**
	**(*n* = 1765)**	**(*n* = 8)**	
**Demographics**			
Sex (male)	1,091 (62%)	6 (75%)	0.72
Age at diagnosis, years (median, IQR)	3.1 (1.7–5.3)	4.7 (2.1–5.3)	0.81
Less than 1 year old	218 (12%)	0 (0%)	0.61
Greater than 9 years old	130 (7%)	0 (0%)	1.00
**Classic Kawasaki disease clinical signs**			
Number of days of fever pre–diagnosis	6 (5–8)	5 (5–6)	0.20
Incomplete Kawasaki disease	483 (29%)	4 (50%)	0.24
Bilateral conjunctival injection	1,433 (88%)	4 (50%)	0.01
Oral changes	1,424 (87%)	7 (88%)	1.00
Extremity changes	1,250 (77%)	6 (75.0%)	1.00
Polymorphous skin rash	1,436 (88%)	8 (100%)	0.61
Cervical lymphadenopathy	962 (59%)	3 (38%)	1.00
**Laboratory investigations**			
Albumin (g/L)	35 ± 6	29 ± 5	0.01
Alanine transaminase (U/L)	28 (16–67)	21 (11–29)	<0.001
Aspartate transaminase (U/L)	37 (27–55)	30 (28–52)	0.04
C-reactive protein (mg/L)	35 (28–47)	35 (31–202)	0.32
Erythrocytes sedimentation rate (mm/h)	68 (41–95)	104 (62–120)	0.08
Hematocrit	0.334 (0.311–0.358)	0.322 (0.266–0.329)	0.11
Hemoglobin (g/L)	113 ± 13	102 ± 17	0.13
Lymphocytes (10^9^ cells per L)	2.6 (1.6–4.2)	1.9 (1.0–3.6)	0.09
Platelets (10^9^ cells per L)	355 (267–462)	408 (315–546)	0.54
Red blood cells (10^12^ cells per L)	4.2 ± 0.5	3.9 ± 0.4	0.14
White blood cells (10^9^ cells per L)	13.0 (9.3–16.9)	17.0 (15.1–20.6)	0.03
**Treatment**			
Multiple intravenous immunoglobulin	221 (14%)	6 (75%)	<0.001
Intravenous steroids	115 (7%)	7 (88%)	<0.001
Oral steroids	56 (3%)	5 (63%)	<0.001
**Complications**			
No coronary artery aneurysms (z-score <2.5)	1,271 (86%)	6 (75%)	0.32
Small coronary artery aneurysms (z-score 2.5–5.0)	125 (9%)	2 (25%)	0.14
Large coronary artery aneurysms (z-score 5.0–10.0)	40 (3%)	0 (0%)	<0.001
Giant coronary artery aneurysms (z-score >10)	43 (3%)	0 (0%)	<0.001
Macrophage activation syndrome	16 (1%)	1 (13%)	0.07

* *Data are reported as frequencies with percentages, means with standard deviation or median with interquartile range*.

† *Obtained from comparison between patients from the KD cohort and KD patients with a co-diagnosis of sJIA*.

*KD, Kawasaki disease; sJIA, systemic idiopathic arthritis; KD/sJIA, Kawasaki disease patients with a co-diagnosis of systemic juvenile idiopathic arthritis*.

KD patients had higher alanine transaminase (median of 28, IQR 16–67 U/L, vs. 21, IQR 11–29 U/L) and aspartate transaminase (median of 37, IQR 27–55 U/L, vs. 30, IQR 28–52 U/L), while KD/sJIA patients had lower albumin (mean of 29 ± 5 vs. 36 ± 6 g/L) and higher white blood cell count (median of 17.0, IQR 15.1–20.6 × 10^9^/L, vs. 13.0, IQR 9.3–16.9 × 10^9^/L) at diagnosis.

A significantly greater proportion of KD/sJIA patients received more than one dose of intravenous immunoglobulin (IVIG) and steroids.

### KD/sJIA Case Summary

**Figure 1:**
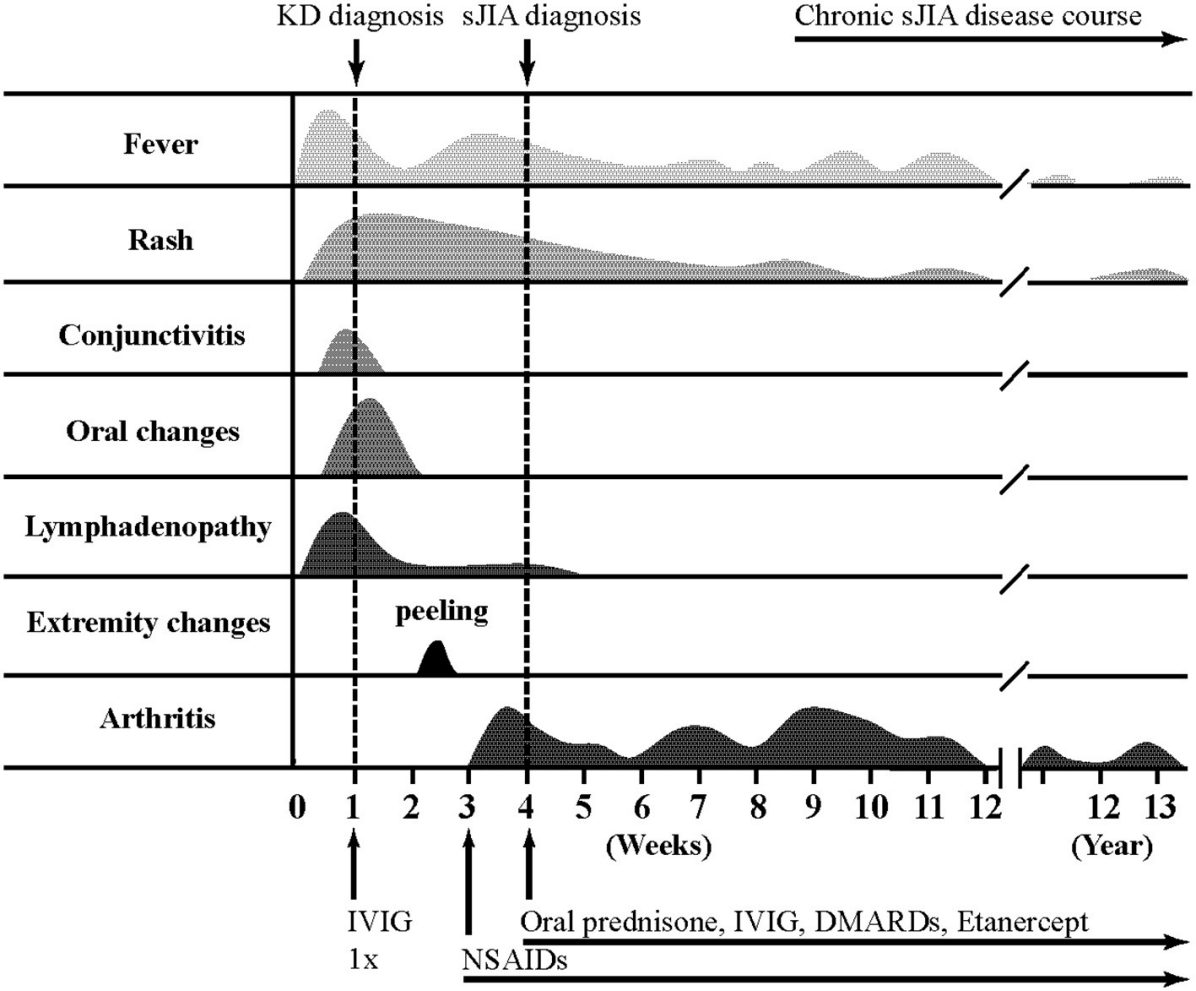
Typical disease course of Kawasaki disease-systemic juvenile idiopathic arthritis.

Detailed clinical features of all patients are reported in Table 2 and Figure 1 illustrates a typical disease course of a KD-sJIA patient.

**Table 2:** Clinical characteristics of KD/sJIA patients.

**Clinical characteristic**	**Case 1**	**Case 2**	**Case 3**	**Case 4**	**Case 5**	**Case 6**	**Case 7**	**Case 8**
Sex	Male	Female	Male	Male	Female	Male	Male	Male
Age at KD diagnosis	1 y, 11 mo	1 y, 11 mo	2 y, 3 mo	4 y, 5 mo	5 y, 0 mo	5 y, 3 mo	5 y, 6 mo	7 y, 3 mo
**Kawasaki disease**								
Days of fever at diagnosis	6	12	5	5	12	6	5	5
KD criteria fulfilled in addition to fever	3/5	3/5	4/5	3/5	3/5	5/5	5/5	4/5
Bilateral conjunctival injection			+		+	+	+	
Oral changes	+		+	+	+	+	+	+
Extremity changes	
Acute	+	+	+	+			+	+
Subacute						+		
Polymorphous skin rash	+	+	+	+	+	+	+	+
Cervical lymphadenopathy		+				+	+	+
Treatment received for KD
Total doses of IVIG (2g/kg)	2	2	2	1	2	1	2	2
Total methylprednisolone pulses	3	4	1	3	3	**–**	3	3
Oral steroids	+	+	+	+				+
Coronary artery dilatation	+	+		+	+			+
Complications		MAS						
**Systemic juvenile idiopathic arthritis**								
Time between KD diagnosis and presumptive diagnosis of sJIA ^°^	22 d	8 d	24 d	30 d	52 d	23 d	90 d	18 d
Time between KD diagnosis and onset of arthritis	24 d	0 d	24 d	0 d	52 d	23 d	2 d	125 d
sJIA criteria fulfilled in addition to fever and arthritis	2/4	4/4	4/4	3/4	3/4	3/4	2/4	3/4
Evanescent rash	+	+	+	+	+	+	+	+
Generalized lymphadenopathy	+	+	+			+	+	+
Hepatomegaly and/or splenomegaly		+	+	+	+	+		
Serositis^†^		+	+	+	+			+
Duration of fever after sJIA diagnosis, mo	8	3	3	7	2	72	2.5	10
Treatment received for sJIA	
NSAIDs	+	+	+	+	+	+	+	+
Oral prednisone	+	+	+	+		+	+	+
DMARDs						+		
Biologics	Anakinra		Canakinumab			Etanercept		
Other						IVIG		IVIG
Length of follow-up	17 mo^§^	12 mo^*^^‡^	18 mo^*^	10 y^§^	2 y^*^^‡^	13 y^*^	11 y^§^	6 y^§^
Disease course	monocyclic	undetermined	undetermined	monocyclic	polycyclic	chronic	monocyclic	chronic

*KD/sJIA, co-diagnosis of Kawasaki disease and systemic juvenile idiopathic arthritis; KD, Kawasaki disease; IVIG, intravenous immunoglobulin; MAS, macrophage activation syndrome; sJIA, systemic juvenile idiopathic arthritis; NSAIDs, non-steroidal anti-inflammatory drugs; DMARDs, disease modifying anti-rheumatic drugs; y - year(s); MO, month(s); D, day(s)*.

+ *Indicates that the symptom was present or treatment was given; – indicates that treatment was not utilized*.

° *Presumptive diagnosis of sJIA before the criterion of 6 weeks of arthritis was met*.

† *All patients had pericarditis and two patients had also pleural effusion*.

* *Indicates patient was on medication at the last clinic visit*.

§ *Indicates patient was off medication at the last clinic visit*.

‡ *Indicates patient was lost to follow-up*.

**Patient 1** is a 23-month-old male who presented with 6 days of fever and 3/5 KD diagnostic criteria with coronary artery (CA) dilatation seen on echocardiography. He was treated with IVIG twice and high dose acetylsalicylic acid (ASA), but remained febrile. He improved after high dose intravenous methylprednisolone pulses (IVMP) and was discharged with a tapering dose of prednisone. The CA dilatation resolved by the 6 week echocardiography follow-up. Two weeks later he developed spiking fevers and arthritis leading to suspicion of sJIA. Recurrent fever and highly elevated inflammatory markers persisted and ultimately he responded well to Anakinra (IL-1 receptor antagonist). Seventeen months post-diagnosis of sJIA, he was in clinical remission and off medication.

**Patient 2** is a 23-month-old female who presented with 12 days of fever and 3/5 diagnostic criteria for KD plus arthritis and CA dilatation on echocardiography. Treatment of incomplete KD consisted of two doses of IVIG and high dose ASA with no clinical improvement. She progressed to have polyarthritis, developed serositis (pleural and pericardial) and her disease evolved into biopsy-proven MAS. She was then diagnosed with sJIA. After high dose IVMP she defervesced and improved clinically, requiring oral steroids for ongoing rash and arthritis. The CA dilatation had resolved by the 6 weeks. One-year post-diagnosis of sJIA, her symptoms were well-controlled with a low dose of prednisone.

**Patient 3** is a 27-month-old male who presented with 5 days of fever and 4/5 KD criteria and pericardial effusion on echocardiography. He received IVIG twice and high dose ASA but remained febrile and was given high dose IVMP followed by oral prednisone with good response and resolution of symptoms. Several weeks after the KD diagnosis, fever recurred with rash and arthritis and consequently he was diagnosed with sJIA. Canakinumab (anti IL-1β) was started for ongoing inflammation. Eighteen months post-diagnosis of sJIA he was in clinical remission on medication.

**Patient 4** is a 4-year-old male who presented with 5 days of fever and 3/5 KD criteria together with clinical signs of congestive heart failure and arthritis. He was treated with high dose ASA, IVIG once and IVMP with resolution of his inflammation. His echocardiography showed pericardial effusion, myocardial dysfunction and CA dilatation. A month after KD diagnosis he again developed rash and arthritis and was diagnosed with sJIA. The CA dilatation had resolved at 6 weeks echocardiography follow-up. Ten years post-diagnosis of sJIA, he was in clinical remission and off medications.

**Patient 5** is a 5-year-old female who presented with 12 days of fever and 3/5 KD features with CA dilatation and pericardial effusion on echocardiography. She was treated with IVIG twice and high dose ASA with minimal response, but improved after high dose IVMP. The CA dilatation had resolved at 6 weeks. Two months later she was diagnosed with sJIA due to persistent fever and arthritis. She re-presented 2 years later with febrile episodes, rash, arthritis and hepatomegaly and was treated for sJIA flare.

**Patient 6** is a 5-year old male who presented with 6 days of fever and 5/5 KD features. Echocardiography showed no abnormalities. He was treated with IVIG once and high dose ASA, but remained febrile. He continued to have high fevers and on day 15 of his illness he developed desquamation of the skin of his fingers. Subsequently he developed arthritis and despite indomethacin, he continued to have intermittent fevers, rash and arthritis and was diagnosed with sJIA. He required anti-TNF therapy due to ongoing systemic symptoms and continued to have destructive chronic arthritis into adult life.

**Patient 7** is a 5-year-old male who presented with 5 days of fever and 5/5 classic features of KD. Echocardiography showed no abnormalities. He developed arthritis of the hip during this hospital stay. Fevers and arthritis were unresponsive to IVIG (twice) and high dose ASA, requiring high dose IVMP to become afebrile. He continued to have intermittent fever, rash and arthritis. After 3 months he was diagnosed with sJIA and the symptoms ceased with indomethacin. Currently, he is in remission and off medication 11 years post-diagnosis.

**Patient 8** is a 7-year-old male who presented with 5 days of fever and 4/5 principal features of KD. He was treated with IVIG twice and received high dose ASA. Echocardiogram revealed pericardial effusion and CA dilatation which resolved by the 6 weeks. He continued to have recurrent spiking fever, rash and serositis leading to the suspicion of sJIA. Inflammatory arthritis was only observed 4 months later when he fulfilled diagnostic criteria for sJIA. At the last visit, 6 years post-diagnosis, he was in clinical remission and off medication.

## Discussion

Both KD and sJIA have their own distinct set of criteria but share similar clinical manifestations at initial presentation. In our KD cohort, 0.5% (8/1765) of children had a co-diagnosis of sJIA; whereas KD preceded sJIA in 7% (8/112) of our sJIA cohort. This is higher than what was reported in 2015 where there as a 0.2% incidence of KD/sJIA among 6,745 KD patients based on patient health information database (3). Patients who fulfilled both KD and sJIA criteria had higher leukocyte count, lower albumin levels, and more refractory to standard KD therapy, suggesting a more severe disease at baseline. Conjunctivitis was a less common presenting symptom in KD/sJIA which is similarly observed in other studies (3).

Fever is a major criterion for both disease entities. In KD fever is self-limiting and, if untreated, lasts for 11 days on average but may continue for several weeks while fever in sJIA can have chronic reoccurrence (4). All our KD/sJIA patients experienced prolonged fever occurring more than 6 months after sJIA diagnosis (Table 2) but not all exhibiting quotidian fever. This is consistent with reports in sJIA that the classic quotidian pattern occurs in <40% of the patients during initial presentation, followed by 27% with intermittent fevers, 15% with bi-daily fevers, and 5% with persistent fevers (4). Rash and lymphadenopathy are also important criteria for both, but with rash being present in all and lymphadenopathy in half of our KD/sJIA patients, none of these criteria are specific for either KD or sJIA. The clinical resemblance is further highlighted by the presence of arthritis, part of the sJIA criteria, in 7.5% of KD patients (5). KD-associated arthritis, however, occurs in the acute phase of the disease that is characteristically self-limiting or resolves within 2 weeks after IVIG treatment. Although the presumptive diagnosis of sJIA was made before the criterion of 6 weeks of arthritis was met, all KD/sJIA patients subsequently met this criterion during follow-up. The most common joint involved was the knee, followed by the ankle and wrist with equal frequency. KD and sJIA share many clinical inflammatory features that appear to be self-limiting in KD and more prolonged in sJIA.

In addition to arthritis, serositis - particularly pericarditis with associated pericardial effusion - can be a common clinical finding in both. Pericardial effusion had been reported in 5–25% of KD patients (6–8). Echocardiography revealed a higher frequency of pericardial effusion in our KD/sJIA patients (63%), three of whom was detected at the time of KD diagnosis and two shortly thereafter. Pericarditis is seen in 10% of sJIA patients (4). It rapidly responds to anti-inflammatory therapy and does not typically recur with good disease control. Similarly, CA lesions in our KD/sJIA patients resolved within 6 weeks and did not recur. Of the eight KD/sJIA patients, two (25%) developed small coronary artery aneurysm and five (63%) had coronary artery dilatation, all of them in children with incomplete KD. The frequency of CA dilatation was 29–42% (9, 10) in other sJIA series, and it was also noted in several case reports in children from 18 months to 6 years old of varying ethnicities (11–14). It is difficult to estimate the true incidence of CA aneurysm in sJIA because of either unreported or varying z-score cut-off values. Although CA dilatation is a key feature of KD, it may not be as unique to this condition as previously thought. Aside from the coronary artery findings, pericardial and myocardial inflammation are well-known entities that are seen in patients with KD and sJIA. These facts may suggest common mechanism that predisposes to cardiovascular morbidity in inflammatory rheumatic disease.

MAS is a serious complication of childhood rheumatic diseases. It is present in 7–22% of sJIA patients (15, 16) and less commonly seen in KD at approximately 1–2% during initial disease presentation (17–19). In our KD/sJIA patients, 13% (1/8) developed MAS shortly after KD diagnosis. Although we have a small number of KD/sJIA patients, which makes it challenging to interpret, other case series have reported similar increase in this unique patient population by as much as 30% (20). This is important since cardiovascular morbidity is reportedly high when KD is complicated by MAS; the incidence of coronary artery abnormalities in particular is 33–100% (17, 21).

The striking association of sJIA and KD with MAS further points to a potentially common immunobiology. All three entities are syndrome complexes with massive immune activation, similar pro-inflammatory cytokine signatures and clinical response to inhibition of common biologic agents (22–26). Interleukin (IL)-1 beta (β) induces fever, is released from keratinocytes in response to stress or injury (27), and is a key mediator of synovial inflammation. It has been shown that serum from sJIA patients have excessive IL-1β production upon stimulation and triggers IL-1β release peripheral blood mononuclear cells of healthy controls (28). Data from genetic and animal studies in KD implicates a pathogenic role of IL-1β in disease development, response to IVIg and cardiac outcomes (29–31). NOD-like receptor family pyrin domain containing 3 (NLRP3) inflammasome transcript levels, protein and cellular response are upregulated in KD compared to healthy controls (30). Although the role of IL-1β in MAS is not entirely clear, IL-1β blockade has been effective in the treatment of MAS (15). The hallmark of MAS pathogenesis is the over-production of pro-inflammatory cytokines (IL-1β) by tissue macrophages, which acts through an autocrine mechanism leading to a vicious cycle of further IL-1β production and exaggerated hyperinflammation. It is worth mentioning that tumor necrosis factor alpha (TNFα) inhibitor has been used as second line agent in KD (32, 33). It shortens the duration of the fever and lowers systemic inflammation markers, but its cardio protective effects in patients with refractory KD has not yet been proven. Similarly, treatment with TNFα inhibitors showed transient but un-sustained improvement in sJIA arthritis, its role in controlling fever and other systemic signs has been unsuccessful (34, 35). These observations are important in both diseases and suggests areas for further investigation into underlying pathobiology.

The newly emerged disease entity during the current severe acute respiratory syndrome coronavirus-2 (SARS CoV-2) pandemic called Pediatric Inflammatory Multisystem Syndrome (PIMS) or Multisystem Inflammatory Syndrome in children (MIS-c) presents with similar clinical features and cardiac complications as that of classic KD (36–38). The cytokine storm with marked elevation in serum inflammatory markers resembling sJIA. Patients are managed with IVIg and corticosteroids, and anti-cytokine therapy with anakinra has been used effectively in severe and refractory cases. All these suggests that PIMS/MIS-c is likely part of this IL-1β disease spectrum as well.

Limitation of our work is its retrospective nature and historical nature of the patient cohort. However, we present clinical information and disease outcomes collected in a large number of KD patients over a span of 20 years. It also allowed us to gather follow-up information in KD/sJIA group, with one patient followed-up to more than 10 years. We recognize that the practice patterns in KD and sJIA management have considerably evolved in the last decade with more aggressive use of biologic drugs like IL-1β and TNFα inhibitors, but the ability to make these observations were facilitated by lack of routine anti-cytokine blockage, supporting the observations and conclusions from this paper.

**Figure 2:**
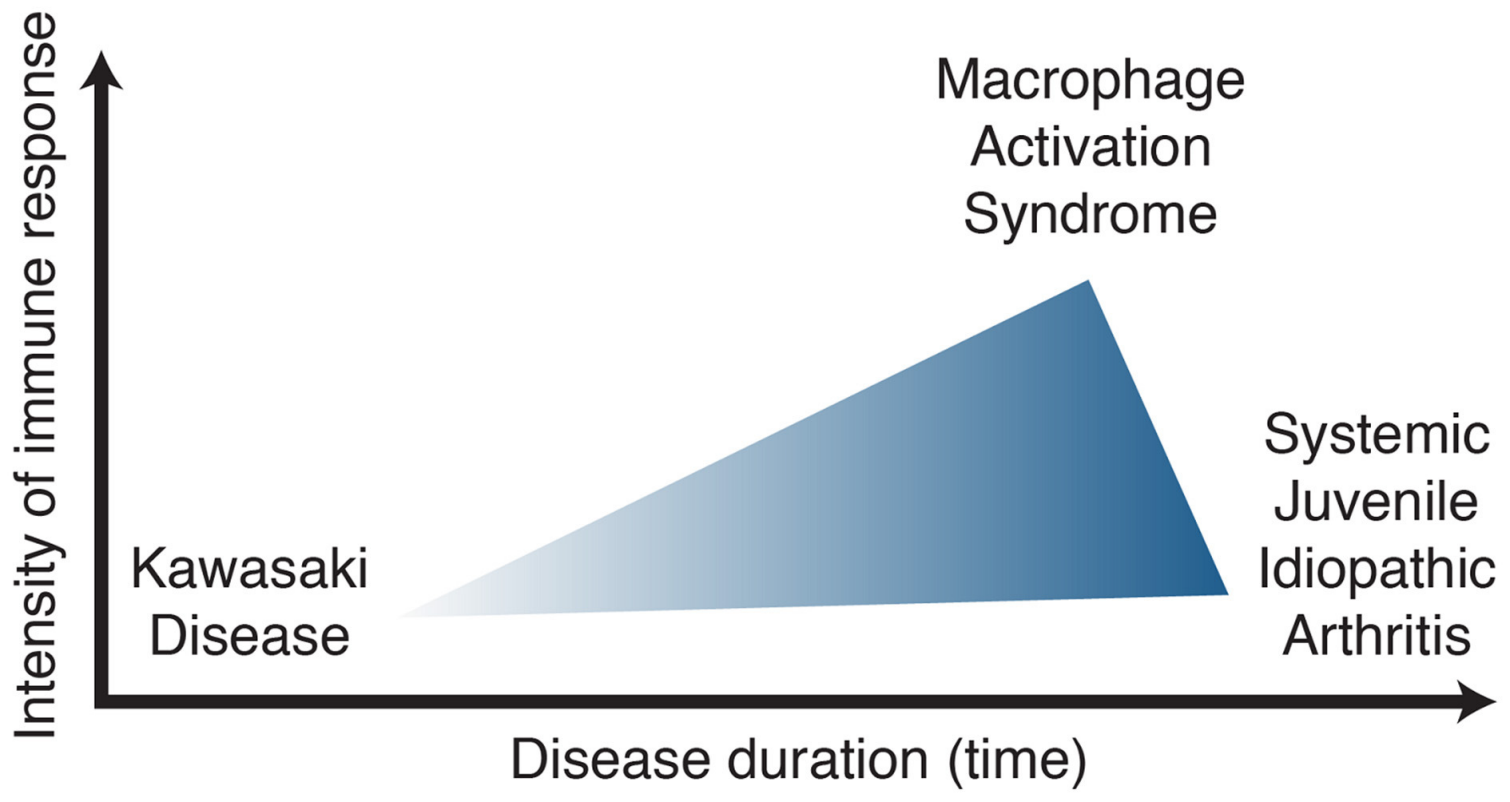
Kawasaki disease, systemic juvenile idiopathic arthritis, macrophage activation syndrome spectrum of disease.

In summary, patients who meet KD criteria should be managed appropriately as such. Prolonged, recurrent fever and rash despite KD treatment should raise suspicion for other systemic inflammatory disorder such as sJIA, especially if arthritis is present. CA dilatation can occur in both disease entities and cannot be used as a sole clinical evidence to differentiate KD and sJIA. Lessons learned from one disease may lead to opportunities for improvement in management. For example, IL-1β blockade in sJIA and MAS may benefit a subset of KD patients with persistent elevation in IL-1β despite IVIg therapy. Alternatively, coronary arteries should be assessed in sJIA patients, and if present, may have implications for therapy, learning from that in KD-associated cardiac disease. Anti-IL-1β treatment may be an effective and more targeted therapy to prevent coronary artery damage in KD, however, more clinical studies are required to know whether it benefits outweigh the risk if used early in those with refractory KD or those with MAS features. Clinical similarities and clues regarding the IL-1β pathway in human and mouse studies suggest common immune biology underlying KD, sJIA and MAS. Thus, we hypothesize that KD, sJIA and MAS are part of the same disease spectrum with the distinguishing features being the intensity and duration of the immune response (Figure 2). Further understanding of the immune biology underlying these syndrome complexes will provide better tools to define, manage and improve outcomes of affected children.

## Data Availability Statement

The original contributions generated for the study are included in the article/supplementary material, further inquiries can be directed to the corresponding author/s.

## Ethics Statement

The studies involving human participants were reviewed and approved by The Hospital for Sick Children Research Ethics Board. Written informed consent from the participants' legal guardian/next of kin was not required to participate in this study in accordance with the national legislation and the institutional requirements.

## Author Contributions

EG participated in and reviewed data collection, revised and critically reviewed the manuscript, and approved the final manuscript as submitted. MvV coordinated and supervised data collection, drafted the initial manuscript, and approved the final manuscript as submitted. CM carried out the initial analyses, reviewed the manuscript, and approved the final manuscript as submitted. RS and BM supervised data collection, critically reviewed the report and approved the final manuscript as submitted. RY conceptualized the study, supervised data collection, critically reviewed and revised the manuscript and approved the final manuscript as submitted. All authors contributed to the article and approved the submitted version.

## Conflict of Interest

RS provided consultation for Sobi and Novartis. The remaining authors declare that the research was conducted in the absence of any commercial or financial relationships that could be construed as a potential conflict of interest.
